# Serological Markers Suggest Heterogeneity of Effectiveness of Malaria Control Interventions on Bioko Island, Equatorial Guinea

**DOI:** 10.1371/journal.pone.0025137

**Published:** 2011-09-27

**Authors:** Jackie Cook, Immo Kleinschmidt, Christopher Schwabe, Gloria Nseng, Teun Bousema, Patrick H. Corran, Eleanor M. Riley, Chris J. Drakeley

**Affiliations:** 1 Department of Immunology and Infection, London School of Hygiene and Tropical Medicine, London, United Kingdom; 2 Department of Infectious Disease Epidemiology, London School of Hygiene and Tropical Medicine, London, United Kingdom; 3 Medicinal Care Development International, Silver Spring, Maryland, United States of America; 4 Ministry of Health and Social Welfare, Malabo, Equatorial Guinea; Menzies School of Health Research, Australia

## Abstract

**Background:**

In order to control and eliminate malaria, areas of on-going transmission need to be identified and targeted for malaria control interventions. Immediately following intense interventions, malaria transmission can become more heterogeneous if interventions are more successful in some areas than others. Bioko Island, Equatorial Guinea, has been subject to comprehensive malaria control interventions since 2004. This has resulted in substantial reductions in the parasite burden, although this drop has not been uniform across the island.

**Methods/Principal Findings:**

In 2008, filter paper blood samples were collected from 7387 people in a cross-sectional study incorporating 18 sentinel sites across Bioko, Equatorial Guinea. Antibodies were measured to *P. falciparum* Apical Membrane Antigen-1 (AMA-1) by Enzyme Linked Immunosorbent Assay (ELISA). Age-specific seropositivity rates were used to estimate seroconversion rates (SCR). Analysis indicated there had been at least a 60% decline in SCR in four out of five regions on the island. Changes in SCR showed a high degree of congruence with changes in parasite rate (PR) and with regional reductions in all cause child mortality. The mean age adjusted concentration of anti-AMA-1 antibodies was mapped to identify areas where individual antibody responses were higher than expected. This approach confirmed the North West of the island as a major focus of continuing infection and an area where control interventions need to be concentrated or re-evaluated.

**Conclusion/Interpretation:**

Both SCR and PR revealed heterogeneity in malaria transmission and demonstrated the variable effectiveness of malaria control measures. This work confirms the utility of serological analysis as an adjunct measure for monitoring transmission. Age-specific seroprevalence based evidence of changes in transmission over time will be of particular value when no baseline data are available. Importantly, SCR data provide additional evidence to link malaria control activities to contemporaneous reductions in all-cause child mortality.

## Introduction

Serological markers have historically been used for evaluating the effects of malaria control interventions [1], [2], [3]. These studies demonstrated a population level decline in antibody prevalence and titre in areas where successful interventions had been put in place. However, serological evaluation fell out of favour due to methodological inconsistency and interpretational difficulties [4]. Recently, serological markers have once again been used to establish estimates for transmission intensity [5], [6], [7], [8] and the availability of specific recombinant antigens for detection of antibodies to both *P.falciparum* and *P.vivax* have increased the sensitivity and specificity of these measures.

In sub-Saharan Africa, where the majority of the malaria burden lies, integrated treatment and vector control programmes have had a considerable impact on reducing malaria prevalence [9], [10], [11], [12], [13], [14]. The Bioko Island Malaria Control Project (BIMCP) was launched in 2004 on the island of Bioko, Equatorial Guinea; Bioko has historically been hyperendemic for malaria, with reported annual entomological inoculation rates (EIRs) of more than 250 and 750 infectious bites per person per year by *An. gambiae* and *An. funestus* respectively [15]. A comprehensive programme of interventions based on vector control and treatment was introduced island-wide, beginning in 2004. The programme was evaluated through annual entomological and parasitological surveys, as well as monitoring and recording of clinical malaria cases. The entomological data showed that the abundance of *Anopheline* mosquitoes dropped 10 fold in the two years following the start of the programme, accompanied by a sharp decline in sporozoite prevalence [16]. Parasitological surveys suggest that the interventions have been extremely successful in some areas, and moderately successful in others [17]. All-cause child mortality across the island has reduced by two-thirds since the start of the programme [18].

A serological investigation was undertaken alongside parasite prevalence and under 5 mortality surveys in 2008, four years after the introduction of malaria control measures in Bioko. The objective of the serological study was to compare changes in malaria exposure defined by *P.falciparum* specific antibody responses with changes in parasite prevalence and changes in all-cause child mortality in response to comprehensive malaria control interventions. This paper describes the congruence of serological measures of exposure with the more conventional parasite rate and demonstrates how serological measures can be used as a monitoring tool in areas where a sharp reduction in transmission intensity has occurred. The study also shows how estimates of serological conversion rates (SCR) and age-adjusted antibody concentrations complement other measures of transmission intensity and can be used to investigate spatial and temporal variation in transmission in response to control activities.

## Methods

### Ethics statement

Ethics approval for this study was granted by the Equatorial Guinea Ministry of Health and Social Welfare. Written, informed consent was obtained from all heads of household prior to the survey.

### Study area

Bioko Island, Equatorial Guinea, located approximately 30 miles west of Cameroon, is 70 km long and 30 km wide, has a mountainous central peak and a population of approximately 250,000 people, the majority of whom live in the flatter, coastal regions of the island. Mean annual rainfall is approximately 2000 mm/year with a peak towards September and October. Bioko has experienced rapid economic growth since the discovery of offshore oil and gas, resulting in a marked increase in standards of living since 2000 [18].

### Interventions

The first five year phase of the BIMCP has been described fully elsewhere [19]. Briefly, a single round of pyrethroid indoor residual spraying (IRS) was introduced in August 2004, followed by bi-annual IRS with carbamate, effective case management (initially using artesunate plus sulphadoxime pyremethamine (SP), then artesunate plus amodiaquine) and intermittent preventative treatment of pregnant women (IPTp) with SP in February 2005. Anti-malarial treatment with artemisinin combination therapies (ACTs) for children under 15 years of age was provided free of charge at all health facilities. A programme of education and communication was established to promote adherence to interventions, capacity development and community participation. In October 2007, long lasting insecticide treated bednets (LLIN) were distributed to cover all sleeping areas. At the time of the 2008 survey, 8 rounds of IRS had been completed.

### Monitoring and evaluation

Malaria Indicator Surveys (MIS) have been conducted annually since 2004 [18]. Households in 18 sentinel sites covering most of the island were randomly sampled from census lists. Although the sentinel sites have not been selected probabilistically, the surveys are representative of Bioko since there are very few areas that are not part of a sentinel site. The surveys included a questionnaire administered to householders to obtain information on household spraying, illness history and attitudes towards, and compliance with, interventions. Children under 15 were tested for malaria parasitaemia using malaria rapid diagnostic tests (RDT) (ICT Diagnostics), detecting *P. falciparum* Histidine Rich Protein-II (HRP-II) and aldolase antigen common to other plasmodial species. These RDTs had previously been shown to be 80% (70–87%) sensitive (when compared with PCR results) with a specificity of 92% (81–98%) [19]. In 2004 and 2008, women of reproductive age from survey households were asked about the history of their previous live births using the standard child mortality module of the Demographic and Health Surveys questionnaire [MEASURE DHS, Macro International Inc., Calverton, MD 20705 USA. Demographic and Health Surveys. Available: http://www.measuredhs.com/aboutsurveys/dhs/questionnaires.cfm#3]. In 2008, filter paper blood spots were collected from all members of each household for use in the serological evaluation reported in this study.

### Laboratory methods

Filter paper blood spots were stored desiccated at 4°C short term and at −20°C for longer term. Antibodies were eluted from bloodspots in 0.5 ml deep well plates (Costar), as described previously [20]. Samples were diluted to a serum dilution equivalent of 1/2000 and antibodies to *P. falciparum* apical membrane antigen -1 (*Pf*AMA-1) (3D7) were detected by ELISA, as previously described [20]. Antibody data were collected as optical density (OD) units and converted to arbitrary titres using a standard curve based on dilutions of hyperimmune serum on each assay plate.

### Statistical methods

**Figure 1:**
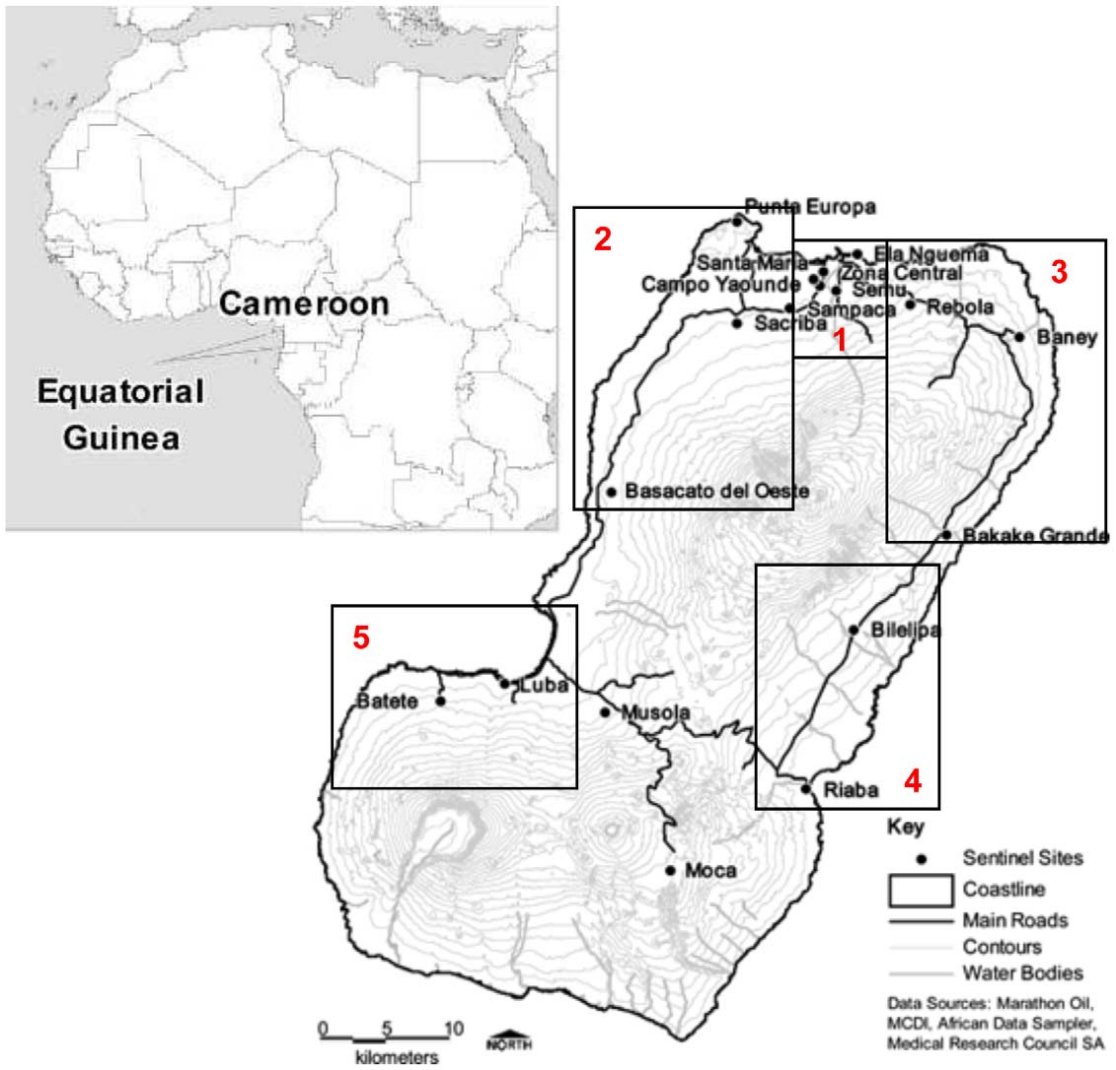
Location of the 18 sentinel sites used for monitoring and evaluation of the BIMCP. Source: Kleinschmidt et al (2007) [17]. Regions are highlighted in boxes. 1- Malabo 2- North West 3- North East 4- South East and 5-South West.

The 18 sentinel sites were designated as the primary sampling units. For the purpose of analysis data were grouped by five geographical regions based on demarcation defined at baseline in 2004 for the purpose of evaluating the interventions [19] (Figure 1).

Maximum likelihood methods were used to define distributions of negative and positive antibody titres; a cut-off value for seropositivity - defined as the mean plus 3 standard deviations of the distribution of negative titres - was then determined using a mixture model [6]. Seroprevalence was calculated as the proportion of samples with an antibody titre above this cut off. The seroconversion rate (SCR), i.e. the rate at which individuals change from being seronegative to seropositive, represents exposure to malaria over time and is analogous to the force of infection [5]. SCR was estimated using catalytic conversion models, assuming a binomial error distribution [21] to fit data on seroprevalence by year of age. The model calculates the SCR (or λ), and the seroreversion rate (ρ; i.e. the rate at which seropositive individuals revert back to seronegativity). Both λ and ρ were allowed to vary in the model to produce optimum fits. Seroprevalence curves were plotted using observed data for 10 age groups, at centiles of the data, plotted at the median age in each centile. The fitted values and 95% confidence limits were also plotted. The resulting λ is presented for each geographical region.

Changes in malaria transmission intensity can be inferred from a discontinuity in the seroprevalence curve [6]. A model allowing for a change in the force of infection at a particular time was used, as previously described [6]. Profile likelihood plots were used to determine the most likely time that a change in transmission intensity occurred. A fall in antibody titres is likely to be detected prior to a change in seroprevalence as the former measure is more sensitive to small changes in transmission. To examine whether changes in antibody titre also reflect recent changes in transmission intensity in a similar manner to changes in antibody prevalence, median antibody titres were also stratified by age.

A method for calculation of under 5 mortality rates using birth history data has been previously reported [18]. By means of proportional hazards regression analysis, hazard ratios for under five mortality for the intervention period relative to pre-intervention were calculated. For the purpose of this study the mortality hazard ratio was calculated for each of the five geographical regions to assess whether changes in child mortality correspond to changes in seroconversion rates, and by implication, force of infection.

### Spatial analysis

The spatial software SaTScan (v7.0.3) was used to detect spatial clusters of infected individuals and higher antibody titres [8], [22]. The Bernoulli model was used to identify areas with higher than expected proportions of RDT positive individuals. Clusters (p<0.05) were assessed based on 999 Monte Carlo simulations to determine the probability of observed prevalence of RDT positives being due to chance relative to expected prevalence of RDT positives under the null hypothesis of no clustering. Circles were restricted to 5 km radius (allowing for maximum mosquito flight distances from breeding sites) with no central overlap with other clusters. The expected number of cases for each circle was estimated and compared to the observed number of cases.

For the detection of spatial clusters in PfAMA-1 antibody responses, the titres were first log10 transformed and then adjusted for age. Loess lines (not shown) were visually assessed to determine the age(s) at which the relationship between antibody titre and age became non-linear. For this dataset, this was determined to be age 20. The residuals from linear regression (log PfAMA titre regressed against age in years, performed in STATA (v.10)) were used to determine whether antibody responses were higher or lower than expected for any given age assuming a homogeneous distribution of risk. Residuals less than zero represent individuals whose responses were lower than average for their age group whilst residuals above zero represent individuals whose responses were higher than average. This method has previously been used for determining clusters of high antibody titre to malaria antigens [8], [23]. The ‘Gaussian model’ for continuous data was used in SaTScan to detect clusters of high responses, using the same constraints as were set for the detection of parasite prevalence clusters. The results were subsequently mapped using ArcGIS (v.9.2) with all significant clusters circled on the map. Residuals for each individual result were scanned in SaTScan; the mean residual per household was plotted on the maps.

## Results

Demographic and serological data for the 2008 survey are summarised in Tables 1 and 2 respectively. Seventeen percent (1075/6367) of the population were positive for *P. falciparum* by RDT, with the highest prevalence in children aged between 5 and 15 (26% positive). Fifty two percent of the population were seropositive to PfAMA, with seroprevalence remaining above 60% for all age groups over the age of 15 years.

**Table 1:** Demographic characteristics of the study population.

		% [n]						
		Malabo N = 2328	North West N = 1749	North East N = 1323	South East N = 700	South West N = 588	Other** N = 699	Total N = 7387
**Age (years)**	0–1	14.1 [324]	10.5 [182]	10.4 [137]	12.2 [85]	10.0 [58]	7.5 [52]	11.4 [838]
	1–5	21.1 [458]	18.0 [312]	19.8 [261]	14.6 [102]	16.8 [97]	15.2 [106]	18.6 [1363]
	5–15	26.3 [605]	30.6 [531]	30.1 [396]	21.0 [146]	24.0 [139]	28.7 [200]	27.5 [2017]
	15–90	38.6 [890]	41.0 [712]	39.8 [524]	52.2 [364]	49.2 [285]	48.6 [338]	42.5 [3113]
**Sex**	Female	61.2 [1410]	54.2 [932]	61.1 [805]	55.8 [389]	58.4 [338]	54.8 [382]	58.2 [4256]
**House recently sprayed** 1	Yes	74.2 [1580]	81.2 [1306]	85.6 [1076]	81.7 [519]	89.5 [477]	87.9 [574]	81.2 [5532]
**Slept under ITN** 2	Yes	82.6 [1629]	68.0 [988]	65.8 [797]	63.3 [404]	73.1 [385]	71.4 [449]	72.4 [4652]
**Parasite positive**	Yes	14.8 [300]	27.0 [374]	7.9 [94]	21.7 [135]	18.6 [97]	12.1 [75]	16.9 [1075]

1 - within the previous 6 months.

2 - on the night before the survey.

**Moca and Musola kept separate due to their high altitude.

**Table 2:** Numbers and prevalence for Rapid Diagnostic Test and anti *P.falciaprum* AMA-1 antibody positive individuals for each sentinel site in Bioko.

Group site	Sentinel site	N RDT	% RDT +ve (95% CIs)	N AMA-1	% AMA-1 +ve (95% CIs)
**Malabo**	Campo Yaunde	393	11.5 (7.8–15.1)	414	42.3 (37.3–47.2)
	Central	374	13.9 (9.5–18.3)	403	50.1 (44.9–55.3)
	Ela Nguema	455	15.6 (11.2–20.0)	511	48.7 (43.3–54.2)
	Santa Maria	394	20.3 (15.7–24.9)	418	53.4 (48.0–58.7)
	Semu	422	12.3 (8.9–15.8)	435	49.4 (44.9–53.9)
**North West**	Basacato del Oeste	325	29.5 (22.2–36.9)	346	67.3 (61.6–73.1)
	Sacriba	391	24.6 (18.6–30.5)	488	67.6 (62.7–72.5)
	Sampaca	330	18.8 (12.9–24.6)	403	68.5 (63.9–73.1)
	Punta Europa	368	34.5 (28.3–40.7)	389	73.8 (69.3–78.2)
**North East**	Bakake	351	10.3 (6.6–13.9)	338	44.4 (39.2–49.5)
	Baney	390	9.7 (5.9–13.5)	406	50.7 (45.8–55.7)
	Rebola	482	4.6 (2.3–6.8)	427	44.5 (39.1–49.9)
**South East**	Bilelipa	290	19.3 (13.5–25.1)	309	39.2 (33.1–45.2)
	Riaba	336	23.8 (18.2–29.5)	347	50.4 (44.8–56.1)
**South West**	Batete	324	13.9 (9.7–18.0)	353	47.0 (41.5–52.5)
	Luba	201	25.9 (18.9–32.9)	215	47.9 (41.5–54.3)
**Other**	Moca	288	10.8 (6.3–15.3)	284	29.9 (24.3–35.5)
	Musola	347	13.0 (8.5–17.4)	371	48.3 (43.1–53.4)
	Total	6461	16.8 (15.6–18.0)	6857	52.0 (50.6–53.3)

Parasite prevalence by RDT (all ages) ranged from 5% in Rebola to 35% in Punta Europa (Table 2). Seropositivity to PfAMA-1 ranged from 30% in Moca to 74% in Punta Europa (Table 2). Similarly, SCRs were approximately ten fold higher in Punta Europa than in Moca (λ = 0.260; 95%CI 0.194–0.348 and λ = 0.030; 95%CI 0.021–0.045 respectively). Site specific parasite prevalence was highly positively correlated with both site specific seroprevalence and SCR (r = 0.7, p<0.01and r = 0.85, p<0.0001, respectively.)

Living in a house that had recently been subjected to IRS was associated with both reduced parasite prevalence (15.8% vs. 18.3%, χ^2^ = 4.2, p = 0.033) and reduced PfAMA-1 seroprevalence (51.0% vs. 54.8%, χ^2^ = 5.6 p = 0.018). More marked reductions in both parasite prevalence and seroprevalence were seen in individuals who reported sleeping under an insecticide treated bednet the night before the survey compared with those who did not report sleeping under a net (parasite prevalence = 14.5% vs. 21.7%; χ^2^ = 41.1, p<0.001; seroprevalence = 50.1% vs. 56.2%; χ^2^ = 17.9, p<0.001).

### Changes in force of infection over time

**Figure 2:**
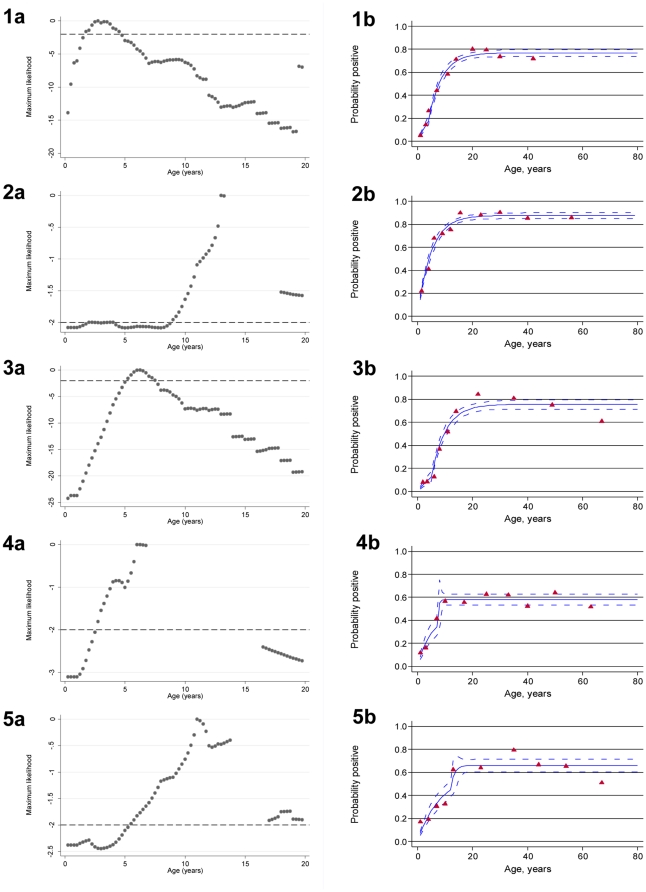
Profile likelihood plots and age-seroprevalence for anti- *P.falciparum* AMA-1 antibody responses by region. Panels 1–5a show univariate profile likelihood to evaluate the time at which seroconversion rates changed for each region 1 to 5 (1-Malabo, 2-North West, 3-North East, 4-South East 5-South West). The broken black line is the 95th percentile of the Chi-squared on 1 degree of freedom below the maximum. The two points at which this line crosses the log-likelihood profile either side of the age at which the likelihood is closest to 0 are used to determine an approximate 95% confidence interval for the time since the change. Panels 1–5b show age seroprevalence plots for anti- *P.falciparum* AMA-1 antibody responses fitted by maximum likelihood with a two forces of infection for each region 1–5. Red triangles represent observed data and blue lines predicted values. Dotted blue lines represent upper and lower 95% CI for the predicted SCR. The steepness of the slope of the line represents the rate of transmission which for regions 1(Malabo), 3(North East), 4(South East) and 5(South West) show a significant reduction in recent years.

For all regions except the North West, the best fit for profile likelihood plots (as estimated by the maximum of the likelihood) indicated a significant discontinuity in λ occurring between 4 to 12 years prior to the 2008 survey (Figure 2). Hence all seroprevalence curves (apart from that for the North West region) fitted significantly better (likelihood ratio test p<0.05) with a model allowing for two values of λ than with a model allowing for only one value of λ. In the Malabo, North East and South Eastern regions, the change in λ was indicative of a highly significant reduction in transmission since interventions began 4 years previously; SCRs were 3–6 fold lower post-intervention than pre-intervention (Table 3). In the South Western region, the best estimates indicated a 10-fold drop in λ occurring approximately 12 years previously (i.e. in 1996), although the confidence intervals around the time of change were large and evidence supporting a model with two SCRs rather than one SCR was rather weak (p = 0.04) (Figure 2).

**Table 3:** Heterogeneity in reductions in transmission: Changes in seroconversion rates for anti *P.falciparum* AMA-1 antibody responses, in *P.falciparum* prevalence and in under five mortality before and after interventions by region.

Region	Time period	AMA-1 SCR (95% CIs)	SCR Ratio Recent/Previous	Pf Prevalence^1^, % (95% CIs)	Prevalence Ratio Post/pre intervention	Under 5 mortality^1^, per 1000 (95% CIs)	Under 5 mortality hazard ratio
**Malabo**	Previous	0.17 (0.14–0.22)	0.30 (p<0.001)	40 (32–51)	0.34(p = 0.001)	110 (52–183)	0.32(p<0.001)
	Recent	0.05 (0.04–0.07)		14 (9–21)		39 (12–84)	
**North West**	Recent	0.18(0.16–0.20)	1(p = 0.163)	47 (43–51)	0.65(p = 0.008)	174 (100–266)	0.44(p = 0.10)
	Previous			30 (21–42)		79 (43–135)	
**North East**	Previous	0.16 (0.13–0.21)	0.17(p<0.001)	41 (31–52)	0.21(p<0.001)	160 (121–209)	0.35(p<0.001)
	Recent	0.03 (0.02–0.04)		9 (5–14)		59 (34–100)	
**South East**	Previous	1.9 (0.04–88)	0.048(p = 0.010)	54 (38–69)	0.51(p = 0.004)	185 (118–284)	0.38(p<0.001)
	Recent	0.09 (0.06–0.13)		27 (26–29)		73 (35–148)	
**South West**	Previous	0.72 (0.08–6.5)	0.01(p = 0.040)	40 (31–49)	0.40(p = 0.03)	189 (134–261)	0.32(p<0.001)
	Recent	0.07 (0.05–0.1)		16 (8–29)		68 (33–140)	

1 from 2004 and 2008 surveys respectively.

In support of the observation indicating no change in malaria transmission in the North West region since the introduction of the malaria control programme, current SCR in the North West region (λ = 0.179; 95%CI 0.158–0.204) remained very similar to the pre-intervention SCR in nearby Malabo (λ = 0.174; 95%CI 0.136–0.223) and North East (λ = 0.164; 95%CI 0.126–0.231) regions. Importantly, these temporal changes in SCR correspond closely with changes in both parasite prevalence and all-cause under-5 mortality (Table 3).

**Figure 3:**
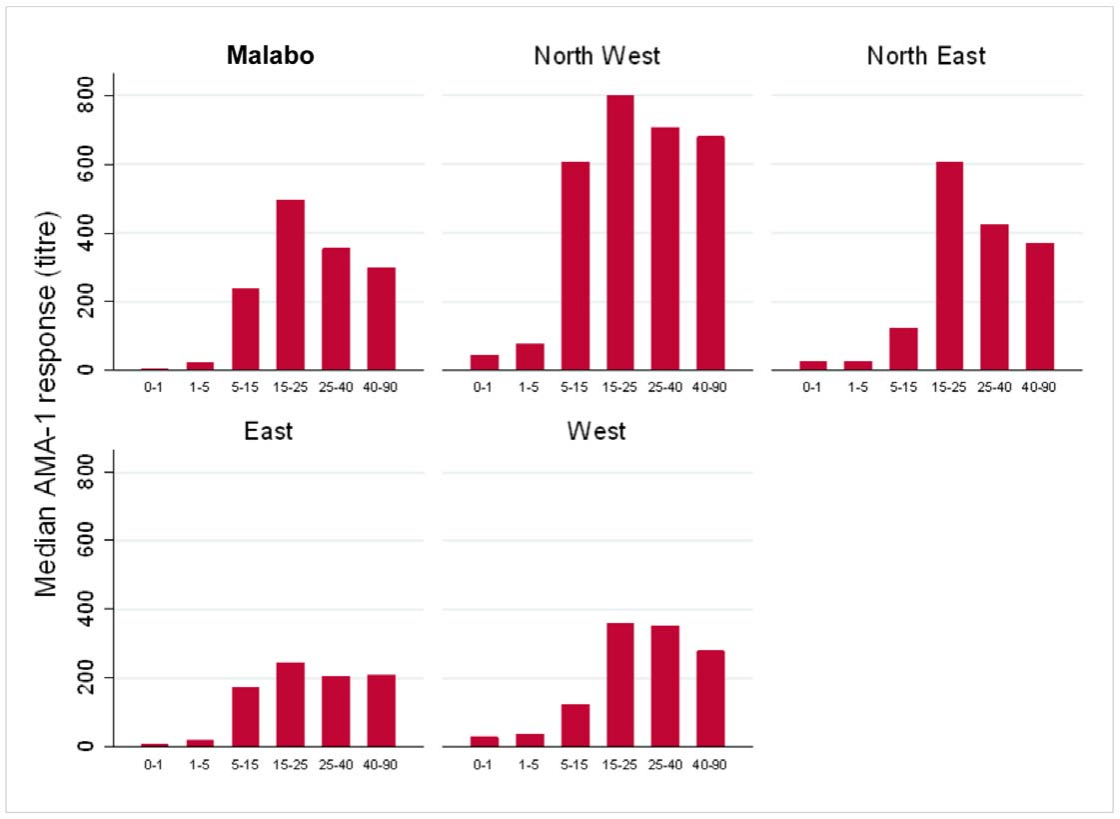
Median anti- *P.falciparum* AMA-1 antibody titres by age group by region.

Figure 3 shows median titres of anti-PfAMA-1antibodies by age group for each region. For all regions, including the North West, PfAMA-1 titres were markedly higher in the age groups above 5 years old; there was, on average, a 9 fold difference (range 2 to 24 fold) in mean titre between the 1–5 year age group and the 5–15 year age group.

### Spatial analysis of malariometric indices

**Figure 4:**
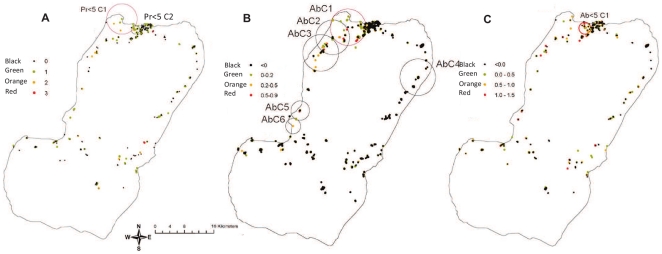
Spatial analyses of parasite positive individuals and anti- *P.falciparum* AMA-1 antibody responses. 4a Spatial distribution of parasite positive individuals under the age of 5 years. This age group was chosen to highlight children who had become infected since interventions began. Colour scale used to denote how many infections were present per household. Black dots are households with no infections in children under 5 years old. SaTScan was used to detect clusters (of no more than 5 km diameter) where there were higher numbers of cases than expected. The primary cluster (most significant cluster) is circled in red, with a secondary clusters circled in black (all p values less than 0.01 for clusters). 4b Spatial distribution of the residual mean log antibody responses to *P.falciparum* AMA-1 in all ages plotted by household. Red circle (AbC1) indicates a cluster of significantly higher than expected antibody responses detected using SaTScan. Secondary clusters (AbC 2–6) are circled in black. 4c Spatial distribution of the residual mean log antibody responses to *P.falciparum* AMA-1 in children under 5 plotted by household. Red circle (Ab<5C1) indicates a cluster of significantly higher than expected antibody responses detected using SaTScan.

Spatial analysis was used to detect clusters of elevated risk of infection based on parasite prevalence in children under 5 years of age and elevated antibody responses (Figure 4). On the assumption that all-age antibody titres represent cumulative exposure over many years whilst antibody titres among children under 5 years of age represent recent exposure, these two analyses are presented separately with household average titre being used to examine clustering of all-age antibody titres (Figures 4B and 4C).

The parasite rates in children under 5 years of age indicated the presence of a primary cluster with a radius of 4.4.km, around Punta Europa (denoted PR<5C1 in figure 4A), in which the parasite rate was four times higher than expected (17 infections observed versus 5 infections expected, p<0.001). There was also a small secondary cluster, contiguous with the primary cluster, in Basacato del Oeste (2.1 km radius, 18 cases observed, 6 expected, p = 0.02; Figure 4A).

For antibody responses in all ages, a 5 km radius primary cluster was identified around Punta Europa, with adjacent clusters involving Sampaca and Sacriba, in the North West region (Denoted AbC1 Figure 4B). The household mean age-adjusted log antibody titre was 0.22 inside the clusters and −0.04 outside the clusters (p<0.001). Secondary clusters were seen in Basacato del Oeste (AbC2 & 3, Figure 4B), also in the North West region, and in Baney and Bakake in the North East (AbC4, Figure 4B all p<0.01). By comparison, analysis of antibody titres in children under 5 years of age (Figure 4C) identified only one small significant cluster (radius 1.6 km and incorporating mainly individuals from Punta Europa but also several from Santa Maria and Central) where the household under 5 mean log titre was 0.56 inside the cluster and −0.06 outside the cluster (p<0.001). The significant clusters for parasite cases and higher antibody titres in children under 5 years of age, overlapped, as one might expect.

## Discussion

The effectiveness of malaria control programmes may not be uniform across regions, resulting in a heterogeneous impact on malaria transmission intensity. In this study we evaluated the utility of serological markers of malaria exposure for assessing heterogeneity in changes in malaria transmission 4 years after the initiation of a comprehensive malaria control programme on Bioko Island, Equatorial Guinea. Our results confirmed the results of previously published parasite prevalence surveys [17], [18], [19] and indicated that, despite considerable reduction in malaria transmission in most areas, the impact of malaria control in Bioko has been uneven. This is demonstrated by differences in the current force of infection (as determined by SCR) across the island and, more convincingly, by differences in the extent to which the force of infection has been reduced (i.e. change in SCR over time) since the initiation of the control programme.

The seroconversion rates show that malaria exposure decreased in the majority of the sentinel sites between 2004 and 2008. Decreasing exposure results in reduced stimuli for the production of antibodies such that antibody prevalence would be lower than otherwise expected in those born since the start of the intervention. In four of the five regions of Bioko, the significantly reduced SCR in the younger age groups clearly indicates a marked reduction in transmission. Moreover, in the one region in which there was no apparent reduction in SCR - the North West - median antibody titres were much lower among children born since malaria control interventions had been in place than among individuals born prior to the start of control activities. This suggests that – in line with the drop in parasite prevalence and under-5 mortality across the entire island [18] - there has in fact been a reduction in malaria transmission intensity in this region but that the impact of this reduction in transmission has not yet led to a noticeable drop in seroprevalence. This might be expected, especially in areas of previously very high transmission where - even after substantial reductions in transmission – infections may still be sufficiently frequent to induce seroconversion in children and to prevent seroreversion in adults but not frequent enough to sustain high titres of antibodies. A similar effect, of reductions in anti-malarial antibody titre after introduction of vector control, was described in the Garki study [24]. It is therefore likely that a measurable shift from seropositive to seronegative would be preceded by a fall in antibody titres. Changes in antibody titre may reflect less pronounced or shorter term changes in exposure.

Due to the retrospective nature of the study, we cannot directly link under-5 mortality to serological status in individual children. However, the under-5 mortality hazard ratios can be ecologically linked to SCRs because they were derived for the same subregions on the island. Although the confidence intervals for the hazard ratios are wide, as a result of the relatively small samples upon which the mortality estimates for each region were made, the point estimates of hazard ratios by region suggest a significant reduction in under-5 mortality in all regions except the North West. When taken together with the parasite data and antibody titre data, this suggests that the relatively modest reductions in transmission in this region were not sufficient to show a statistically significant impact on mortality. In malaria endemic countries where disease-specific mortality statistics are unavailable, it has been argued that reductions in all-cause child mortality can be attributed to malaria control efforts if improvements are found in steps on the causal pathway between the scaling up of programmatic efforts, and mortality, i.e. if there is evidence of high coverage of interventions, and evidence of a reduction in at least one malaria-specific indicator such as prevalence of infection, then a reduction in all-cause child mortality is likely to be linked to the interventions [25]. This ‘plausibility’ argument is made somewhat more plausible in our study because reductions in seroconversion rates correspond to reductions in under five mortality rates. We would therefore argue that serological surveys be included in the evaluation of large scale interventions to strengthen the evidence for the link between successful interventions and all-cause child mortality.

Parasitological, serological and child mortality measures all suggest that malaria transmission remained high in the North West of Bioko. The effectiveness of malaria control in this region may be enhanced by identifying foci of ongoing transmission which can be targeted for more frequent or concerted attention. The spatial analysis of all-age antibody titres identified several areas where transmission was historically higher than elsewhere - demonstrated by the stability of the antibody response in older individuals. In the North West, these clusters coincide with one significant cluster of high titre antibody responses in children under 5 years of age, indicating persistent high transmission. In contrast, despite significant clusters in adults in the North East, none are detected in children under 5, presumably in response to successful implementation of malaria control in this area. The reasons for the persistence of high transmission in the North West of Bioko have not yet been fully understood. Possible explanations that have been advanced are inadequate IRS coverage in some years, human behaviour typified by outdoor activities during night-time vector biting, outdoor host seeking behaviour of *An.Gambiae* [26] thus rendering interventions less effective, and a greater abundance of breeding sites as a result of the flat terrain compared to other parts of Bioko.

Whilst the observed decline in malaria transmission in most of Bioko coincides with the introduction of intensive malaria control measures, it is likely that other factors such as socio-economic development may have contributed to this decline [18]. The intervention years have also been characterised by somewhat lower average annual rainfall than pre-intervention (1710 mm versus 2160 mm). However it is questionable whether a relatively modest reduction in annual rainfall at such high levels would result in reduced malaria transmission.

Our analyses demonstrate that adding a serological component to malaria indicator surveys provides important additional information. Firstly, since antibody responses integrate exposure over time, serological data from a single cross-sectional survey can provide information on historical changes in transmission. For example, in Tanzania a change in SCR was identified approximately 15 years previously [6] and similar studies were used to confirm malaria elimination on Mauritius [27] and in Greece [28]. In this study in Bioko, the historical transmission intensities (based on SCR) in several of the regions were of a similar magnitude to the current (and historic) SCR in the North West region, suggesting that transmission was relatively uniform across the island prior to implementation of the BIMCP [29]. Secondly, as transmission intensity declines in response to successful control activities, parasitological measures become less informative and more sensitive methods (such as serology and molecular parasite detection) will become increasingly important. Establishing the congruence between parasitological and serological measures of exposure in relatively high transmission settings – as we have done here – will allow a more robust interpretation of serological data from low transmission areas where parasite prevalence indicators are insufficiently sensitive. Thirdly, although parasite prevalence had begun to fall in many areas of Bioko it remained high in some places, which were identifiable from the serological data. Targeting of such transmission foci with carefully planned interventions may be the most effective way to reduce transmission in these districts [29].
